# The Potential Use of Methotrexate in the Treatment of Cutaneous Leishmaniasis: In Vitro Assays against Sensitive and Meglumine Antimoniate-resistant Strains of *Leishmania tropica*

**Published:** 2017

**Authors:** Hossein MAHMOUDVAND, Farnaz KHEIRANDISH, Seyed Reza MIRBADIE, Mohammad Hassan KAYEDI, Tahereh REZAEI RIABI, Abbas Ali GHASEMI, Mehdi BAMOROVAT, Iraj SHARIFI

**Affiliations:** 1. Razi Herbal Medicines Research Center, Dept. of Medical Parasitology and Mycology, Lorestan University of Medical Sciences, Khorramabad, Iran; 2. Dept. of Medical Parasitology and Mycology, School of Medicine, Shahroud University of Medical Sciences, Shahroud, Iran; 3. Leishmaniasis Research Center, Kerman University of Medical Sciences, Kerman, Iran

**Keywords:** Meglumine antimoniate, Resistance, *Leishmania tropica*, Methotrexate, In vitro

## Abstract

**Background:**

The present study aimed to evaluate the effect of methotrexate (MTX) alone and in combination with meglumine antimoniate (MA, Glucantime) against sensitive and MA-resistant *Leishmania tropica* stages in vitro.

**Methods:**

The present study was carried out in 2014 in Leishmaniasis Research Center at School of Medicine, Kerman University of Medical sciences, Kerman, Iran. The effects of MTX alone and along with MA on promastigote and amastigote stages of sensitive (SS) and MA-resistant (RS) *L. tropica* strains have been evaluated using a colorimetric MTT assay and in a macrophage model, respectively. In addition, the inhibitory effect of MTX on the *Leishmania* invasion of murine macrophages was assessed in promastigotes of both strains of *L. tropica*. Sensitive and MA resistant *L. tropica* are referred to those isolates that are responsive or non-responsive to one or two courses of treatment by MA systemically and/or intralesionally, respectively.

**Results:**

The findings of OD and IC_50_ showed that MTX plus MA (SS: 16.1 μg/ml, RS: 39.8 μg/ml) had a higher anti-leishmanial effect than MA (SS: 52.2 μg/ml, RS: 170 μg/ml) or MTX alone (SS: 22.2 μg/ml, RS: 51.4 μg/ml) on promastigotes of both strains of *L. tropica.* The MTX plus MA caused a significant decrease (*P<*0.05) in the mean infection rate (MIR) and the mean number of amastigotes in each macrophage compared with positive control. Infectivity of promastigotes is significantly (*P<*0.05) reduced when it was preincubated with MTX.

**Conclusion:**

This study indicated high potency and a synergistic effect of MTX on MA in inhibiting the growth rateof promastigote and amastigote stages of sensitive and meglumine antimoniate-resistant *L. tropica*. Further works are needed to evaluate the anti-leishmanial effects of MTX on *L. tropica* using a clinical setting.

## Introduction

Leishmaniasis caused by parasitic protozoa of the genus *Leishmania* is an infection consisting of four clinical types including cutaneous, diffused cutaneous, mucocutaneous and visceral forms. Cutaneous leishmaniasis (CL) is identified by continuing nodulo-ulcerative wounds curative spontaneously with scarring (1). CL is considered as main community health and social problem in many regions of the world particularly in the Eastern Mediterranean Region, and approximately all countries of the Middle East (2). In Iran, both epidemiological forms of CL are present; anthroponotic CL (ACL) and zoonotic CL (ZCL) caused by *L. tropica* and *L. major,* respectively (3, 4).

Presently, there is no safe and successful vaccine existing. The control policy efforts are hampered by varied ecology of species of sand fly vector and reservoir host. In fact, the diversity of clinical symptoms and epidemiological types make it hard to apply a single measure, globally (5). In ACL, the effectual control way is timely detection of the cases and early treatment (6). This effort is moderately inadequate because of increase of resistance to antimonials in present use, although the spatial distribution and the degree of resistance to any single drug vary deeply (7).

There are numerous drugs available for the treatment of CL, although “watch and wait” is also advocated due to spontaneous healing which usually occurs after several months. At present, using the first-line drugs including pentavalent antimonials (meglumine antimoniate (MA) and sodium stibogluconate (SSG) exhibited some problems such as prolonged systemic therapy, high toxicity, less effectively against various forms. Moreover, the second-line drugs also have limitations for use because of high cost, prolonged length of therapy and adverse reactions (8–10). For these reasons, development of new drugs or combination therapy for treatment of CL is urgent. Nowadays, wide efforts have also been made to support combination therapy of existing drugs including verapamil, imiquimod, and allopurinolthat showed synergistic effects with meglumine antimoniate in the treatment of CL (11–14).

Methotrexate (MTX) is an antimetabolite and antifolate drug, that significantly inhibits dihydrofolate reductase (DHFR), an enzyme that catalyses the conversion of dihydrofolate to active tetrahydrofolate. Folic acid (folate) is required for the production of the nucleoside thymidine needed for DNA synthesis (15). Currently, MTX has been applied to remedy of various forms of cancers, severe psoriasis, rheumatoid arthritis and ectopic pregnancy (16–21). Reviews have shown anti-parasitic property of MTX on chloroquine-sensitive and multidrug-resistant strains of *Plasmodium falciparum*, pyrimethamine-resistant *P. vivax*, *Echinococcus multilocularis* metacestodes and conjugated with branched polypeptide against *L. donovani* (18, 21–24).

The present study aimed to evaluate the anti-leishmanial effects of MTX alone and in combination with meglumine antimoniate on promastigote and amastigote forms of sensitive and MA-resistant strains of *L. tropica* using colorimetric assay (MTT) and macrophage model, respectively.

## Materials and Methods

### Chemicals

Methotrexate and MTT [3-(4.5-dimethylthiazol-2-yl)-2.5-diphenyl tetrazolium bromide)] was prepared from Sigma-Aldrich (St Louis, MO, USA), penicillin and streptomycin were obtained from Alborz Pharmacy, Karaj, Iran and also meglumine antimoniate was purchased from Rhône (Poulenc, France) and stored at room temperature (25°C) until testing. In addition, RPMI-1640 containing, L-glutamine (2 mM) and fetal calf serum (FCS) were prepared from Gibco (Eching, Germany). All other chemicals and solvents were of analytical grade just prepared in 2014 before performing experiments.

### Parasite culture

Sensitive and MA resistant *L. tropica* are referred to those isolates that are responsive or non-responsive to one or two full courses of treatment by MA systemiclly and/or intralesionally, respectively. Sensitive (MHOM/IR/2002/Mash2) strain of *L*. *tropica* was obtained from the Center for Research and Training in Skin Diseases and Leprosy (Tehran, Iran). MA-resistant strain of *L*. *tropica* was prepared from a CL patient in Bam, southeastern Kerman Province of Iran. This isolate was detected by nested-PCR as *L. tropica* and further identified by conventional PCR for MDR1 gene [8]. Subsequently, the DNA extract was sequenced and recorded in GenBank under HM854717 Accession Number. The parasite was cultured in RPMI-1640, supplemented with penicillin (100 IU/ml), streptomycin (100 μg/ml), and 15% heat-inactivated fetal calf serum (FCS).

### Cell culture

Murine macrophages were collected from male BALB/c mice (6–8 wk old) by injecting 5 ml of RPMI-1640 medium into mouse peritoneal cavity and aspirated macrophages were washed twice and resuspended in RPMI-1640 medium, until testing.

The experimental procedures carried out in this survey complied with the guidelines of the Kerman University of Medical Science (Kerman, Iran) for the care and use of laboratory animals.

### Anti-proliferation effect against promastigotes forms

The ability of MTX alone and in combination with MA to inhibit the proliferation rate on promastigote forms of sensitive and MA-resistant strains of *L. tropica* was assessed by colorimetric cell viability MTT assay using the method (25). Briefly, the promastigotes (10^6^ cells/ml) in the logarithmic growth phase were cultured in complete medium and 100μl of medium was added into a 96-well microtiter culture plate. Then 100 μl of different concentrations of MTX alone (2.5–100 μg/ml), MA alone (2.5–100 μg/ml) and along with MTX (2.5μg/ml) was added to each well and incubated at 24°C for 72 h. In the next step, 10μl of MTT solution (5 mg/ml) was added to each well, incubated at 24°C for 3–4 h. Finally, the absorbance was measured using an ELISA reader (BioTek-ELX800) at 490 nm. The 50% inhibitory concentrations (IC50 values) were calculated by Probit analysis method using the software SPSS 17 for windows (Chicago, IL, USA). Promastigotes were cultured in complete medium with no drug used as control, and complete medium with no promastigote was used as blank. All experiments were repeated in triplicates.

### Effect on intra-macrophage amastigotes of both strains

Murine macrophages collected from male BALB/c mice were used according to the method described earlier (26). Sterile 1cm^2^ cover slips were placed in the wells of 6-chamber slides (Lab-Tek, Nalge Nunc International NY, USA) and then 200 μl of the murine macrophages (10^5^cells/ml) was placed in each well. After 2 h of incubation at 37°C and 5% CO_2_, promastigotes of both strains in the stationary phase were added to each well at a ratio of 10 parasites per macrophage and incubated at the same conditions for 24 h. Excess parasites were then removed by washing with RPMI-1640 medium and infected macrophages were treated with different concentrations of MA or MTX alone (10–200 μg/ml) and various concentrations of MA (10–200 μg/ml) along with 2.5 μg/ml of MTX at 37°C for 72 h. Finally, dried slides were fixed with methanol, stained with Giemsa and studied under a light microscope. Positive and negative controls included infected macrophages with no drugs and non-infected macrophages with no drugs, respectively. Anti-leishmanial effects were evaluated by counting the number of amastigotes in each macrophage by examining 100 macrophages on each coverslip using a light microscope (27). The IC_50_ values (μg/ml) also were calculated by Probit analysis method using the software SPSS 17 (SPSS Inc., Chicago). All experiments were carried out in triplicate similar to promastigote stage.

### Inhibition of infection in murine macrophages

To evaluate the inhibitory effect of MTX on *L. tropica* invasion, promastigotes in the stationary phase were pre-incubated in 2.5 μg/ml of MTX alone and in combination with MA for 2 h at room temperature. Then, they were washed with RPMI-1640 medium and again incubated with murine macrophages for 4 h at the room temperature. After washing, the macrophages were fixed with methanol, stained by Giemsa and studied under a light microscope. The mean infection rate of macrophages was calculated by counting 100 macrophages on each coverslip as compared with control (28).

### Statistical analysis

All data represent the means ± standard deviations (SD) of three independent experiments. Data were analyzed by one-way ANOVA tests and Scheffe Post Hoc tests, using software SPSS 17 for windows (SPSS Inc., Chicago, USA). Moreover, to compare the IC_50_ values of groups and control drug t-test was used and a *P*-value less than 0.05 was considered significant.

## Results

### Anti-promastigote effects

**Fig. 1:**
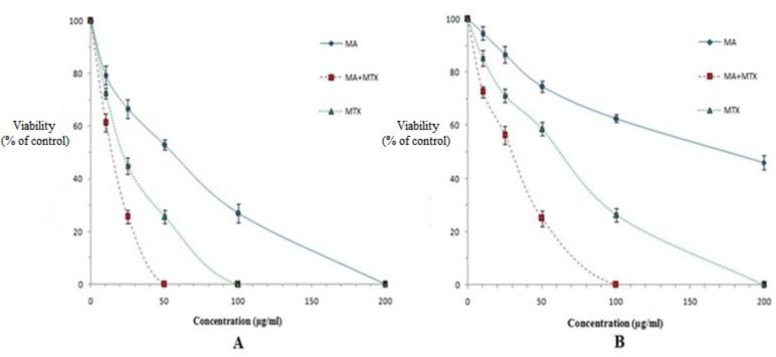
The viability of promastigotes of sensitive (A) and MA-resistant (B) strains of *L. tropica* in the presence of various concentrations of meglumine antimoniate (MA), methotrexate *(*MTX*)* and MTX+MA after 72 h incubation. Data are expressed as the mean ± SD (n = 3).

Anti-proliferation effects of MTX against promastigotes forms of sensitive and MA-resistant strains of *L. tropica* were evaluated by colorimetric cell viability MTT assay. As shown in Fig. 1, MTX, especially in combination with MA significantly (*P*<0.05) inhibited the proliferation rate of promastigotes of both strains and showed a dose-dependent inhibition as compared with MA.

The IC_50_ values of MA for sensitive and MA-resistant strains were 52.2 and 170 μg/ml, respectively. Whereas the IC_50_ values of MTX alone for sensitive and MA-resistant strains were 22.2 and 51.4 μg/ml, respectively. These values are significantly (*P*<0.05) higher than the measured IC_50_ values for various concentrations of MTX along with MA against sensitive and MA-resistant strains of *L*. *tropica* (16.1and 39.8 μg/ml, respectively), indicating less effectivity of MA or MTX alone as compared with combination of MTX+ MA on promastigotes of both strains of *L*. *tropica* (Table 1).

**Table 1:** Comparison of the mean IC_50_ values among various concentrations of meglumine antimoniate (MA), methotrexate *(*MTX*)* and MTX+MA against the growth rate of promastigote and amastigote forms of sensitive and MA-resistant strains of *L. tropica*. Data are expressed as the mean ± SD (n=3)

**Chemicals**	**IC_50_ (μg/ml)**			
	**Amastigote**		**Promastigote**	
	Resistant	Sensitive	Resistant	Sensitive
MA	12.7±2.05	55 ± 2.15	52.2± 2.08	170± 3.17
MTX	8.0±0.5	15.7 ± 2.52	22.2± 1.5	51.4± 3.3
MTX+MA	4.2±1.15	10.8 ± 1.57	16.1± 2.08	39.8± 2.5

### Anti-intramacrophage amastigote effects

The in vitro anti-amastigote effects of MTX were examined by measuring the mean number of amastigotes in each macrophage infected by promastigotes of both strains of *L*. *tropica*.

**Fig. 2:**
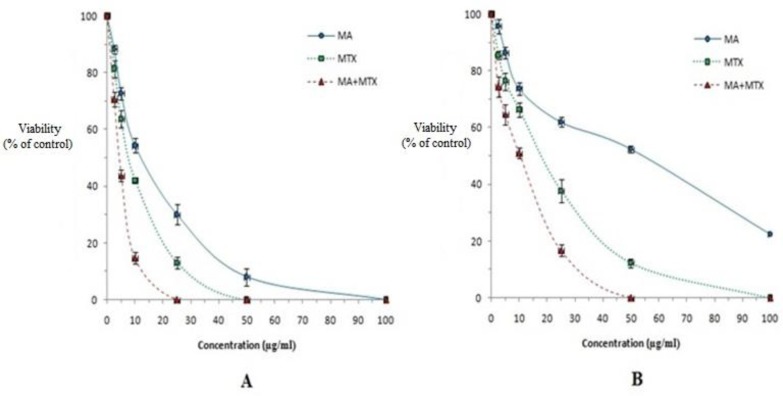
The effect of various concentrations of meglumine antimoniate (MA), methotrexate *(*MTX*)* or MTX + MA on the mean number of amastigotes of sensitive (A) and MA-resistant (B) strains of *L*. *tropica* in each macrophage (amastigotes viability) in comparison with infected macrophages with no treatment as positive control. Data are expressed as the mean ± SD (n = 3).

The results of the mean number of amastigotes in each macrophage showed that MA plus MTX combination efficiently reduced (*P<*0.05) the number of amastigotes in each macrophage in all concentrations when compared with MA or MTX alone (Fig. 2). MTX alone induced more anti-amastigote effect than MA alone against both strains of *L*. *tropica*. The IC_50_ values of MA plus MTX indicated more effective anti-leishmanial effects on amastigote forms of both strains of *L*. *tropica* when compared with MA or MTX alone (Table 1).

### Inhibition of infection in macrophages

Since infectivity is one of the most important pathogenic and biological criteria of *Leishmania* parasites, the effects of MTX alone and in combination with MA on the infectivity of promastigotes of both strains of *L*. *tropica* to murine macrophages were evaluated. In the absence of any drugs, originally ≥75% of the macrophages were infected by promastigotes of sensitive and MA-resistant strains of *L*. *tropica*. In contrast, promastigotes of sensitive strain of *L*. *tropica* treated with MA or MTXalone and MA+MTX in combination were able to infect 28.5%, 48% and 16% of macrophages, respectively. Whereas promastigotes of MA-resistant strain of *L*. *tropica* treated with MA, MTX and MA along with MTX had ability to infect 63%, 53%, and 32% of macrophages, respectively. The infectivity of promastigotes of both *L. tropica* strains significantly *(P<0.05)* reduced with MTX pre-incubation.

## Discussion

Leishmaniasis is one of the most significant public health problems in tropical and sub-tropical areas. The diseases are endemic in 98 countries and nearly affects 12 million people and threatens 350 million people in the world (1). There is currently no successful and protected vaccine against leishmaniasis, the control of this disease depends on rapid case finding and suitable chemotherapy. For several decades, pentavalent antimonials (MA and SSG) are the first option drugs for CL. These drugs have restriction for utilizing because of emergence of resistant parasites, toxicity and long duration of treatment [9, 29, 30]. Therefore, there is an urgent need for development of new effective treatment regimens against different types of leishmaniasis. Recently, combination treatments with MA have been applied for CL with a wide range of drugs that demonstrated their interactive properties accompanied by MA in treatment of CL [11–14]. Other new drugs approved for the oral treatment of ACL and/or ZCL including miltefosine, ketoconazole, itraconazole, allopurinol, dapsone and various physical therapies such as cryotherapy, surgical excision, and heat are also used [29–32].

In this study, anti-leishmanial activity of MTX as an inhibitor of DHFR alone and in combination with MA against sensitive and MA-resistant strains of *L. tropica* has been evaluated. Our finding showed that MTX, especially in combination with MA, has higher anti-leishmanial effects against promastigote and amastigote forms of both strains of *L. tropica* as compared with MA alone. In addition, amastigotes forms in both strains were more susceptible to treatment with MTX alone or along with MA. Of course, the difference in susceptibility of promastigote and amastigote stages in response to various concentrations of MTX is related to their structural, biochemical and morphological features (33).

In the case of anti-parasitic effects of methotrexate, for the first time, the clinical usefulness of MTX against *P. vivax* infection was reported (21). Of 2.5 mg, oral dose of MTX given daily for 3 d completely cleared parasitemia within 48 h, without any side effects. A branched polypeptide-methotrexate conjugate with a polycationic carrier (ALK) could increase the effect of MTX against *L. donovani* infection on in vitro and in vivo (24). In addition, the in vitro combination of MTX with polypeptides was shown to inhibit the growth rate of *L. donavani* stages. In contrast, sub-optimal doses of MTX may lead to development of drug resistance by the parasite in *L. donovani* [34].

The anticancer MTX and trimetrexate (TMX), inhibitors of dihydrofolatereductase (DHFR), were highly potent against pyrimethamine-resistant *P. vivax* and both sensitive and multidrug-resistant strains of *P. falciparum* (18, 23). In addition, MTX alone at low dose or in combination with 5-methyl-tetrahydrofolate (5-Me-THF) could be used to treat *P. falciparum* malaria (19).

In consistent with previous reports, the present experimental study showed that MTX especially in combination with MA, at the low doses and short times have potent anti-parasitic effects against sensitive and MA-resistant strains of *L. tropica*. In contrast to these studies, the cytostatic drugs such as MTX not only did not show parasiticidal or clear parasite static effects on metacestodes of *E. multilocularis*, but also MTX pre-treatment seemed to enhance parasite proliferation (22).

MTX has many advantages such as its half-life, which at low doses is 3–10 h, which is approximately shorter than those of available anti-leishmanial drugs are. Therefore, resistance to this drug is less likely to develop. MTX is taken by oral, intravenous and intramuscular, which is another advantage over many currently anti-leishmanial drugs. The major concern in the use of MTX as treatment of leishmaniasis is its side effects because side effects in long-term and high-dose of MTX use are numerous. However, side effects of MTX in short-term treatment, even at relatively high doses are few and rare [18]. Regarding toxicity effects of MTX studies demonstrated that although MTX blocks folic acid-dependent steps in the synthesis of purines and pyrimidines and limits the proliferation of malignant cells; however, this effect on purine and pyrimidine biosynthesis can also have some toxicity, such as bone marrow suppression and stomatitis. Concomitant administration of folic acid to patients taking methotrexate was showed no difference in therapeutic efficacy of the methotrexate and prevention of methotrexate-mediated toxicity (18, 35). Therefore, administration of folic acid may suppress side effects in high-risk patients. Our finding is in consistent with previous studies, which demonstrated that low doses of MTX in short time are needed to inhibit the parasite. Therefore, the risk of side effects of this drug may be minimized.

## Conclusion

This study demonstrates high potency and a synergistic effect of MTX alone and combined with MA in inhibiting growth of promastigote and amastigote stages of sensitive as well as MA-resistant strains of *L. tropica.* In addition, further clinical studies are required to investigate this synergistic effect on a therapeutic scale.
